# Social Distance Monitor with a Wearable Magnetic Field Proximity Sensor

**DOI:** 10.3390/s20185101

**Published:** 2020-09-07

**Authors:** Sizhen Bian, Bo Zhou, Paul Lukowicz

**Affiliations:** 1 German Research Center for Artificial Intelligence (DFKI), 67663 Kaiserslautern, Germany; bo.zhou@dfki.de (B.Z.); paul.lukowicz@dfki.de (P.L.); 2 Department of Computer Science, University of Kaiserslautern, 67663 Kaiserslautern, Germany

**Keywords:** magnetic field, magneitic sensing, magnetic sensor, proximity sensing, COVID-19, social distancing

## Abstract

Social distancing and contact/exposure tracing are accepted to be critical strategies in the fight against the COVID-19 epidemic. They are both closely connected to the ability to reliably establish the degree of proximity between people in real-world environments. We proposed, implemented, and evaluated a wearable proximity sensing system based on an oscillating magnetic field that overcomes many of the weaknesses of the current state of the art Bluetooth based proximity detection. In this paper, we first described the underlying physical principle, proposed a protocol for the identification and coordination of the transmitter (which is compatible with the current smartphone-based exposure tracing protocols). Subsequently, the system architecture and implementation were described, finally an elaborate characterization and evaluation of the performance (both in systematic lab experiments and in real-world settings) were performed. Our work demonstrated that the proposed system is much more reliable than the widely-used Bluetooth-based approach, particularly when it comes to distinguishing between distances above and below the 2.0 m threshold due to the magnetic field’s physical properties.

## 1. Introduction

The spread of COVID-19 threatens global public health. Besides the effort to explore an efficient vaccine, measures, like lock-down [1,2], mask-wearing [3], and social distancing [4,5], are being taken to slow the spread and prevent severe consequences of the pandemic. As the most crucial step, social distancing is widely explored, aiming to track the contact of a contagious patient: anyone who has been within this distance of the contagious person for a significant amount of time could potentially have been infected. The World Health Organization has declared a social distance of at least 1 m among patients and healthcare workers at inpatient facilities, as well as other spaces, like outpatient facilities, and other communities, like home and public areas [6]. Other organisations also released statement of social distancing with 1.5 m [7,8] or 2 m [9,10].

Today, contact tracing apps that use BT (Bluetooth) proximity sensing have been deployed worldwide to track social distancing. While they have the advantage of being easily deployable, as they require nothing more than a standard smartphone, they tend to suffer under the well-known accuracy and reliability problems [11,12,13]. For everyday consumer applications, the ease of deployment of smartphone-based solutions may out-weight the accuracy problems. However, for other more sensitive settings, such as healthcare, critical industries, or education (opening schools during a pandemic), there has been significant interest in more reliable dedicated proximity sensing solutions. Time of light systems, for example, the Ultra-sound [14], have an inherently higher accuracy, but suffer from the robustness issues in real world environments. In particular, it is prone to multi-path propagation errors. There are also various real world ultrasound sources that can disturb the signal. Capacitive body sensing [15] is a low cost, low power consumption alternative, however it only works reliably when subjects move relative to each other generating dynamically changing signals. It is also prone to noise related to changes in the environment not related to the proximity between users. Other viable methods, including thermal image [16], cellular and GPS (Global Positioning System) [17], and AI-based computer version [18,19,20] are often under privacy policy debate, as the users’ visual appearance or exact location path reveals more information than that needed for mere contact detection.

In this paper, we demonstrate how oscillating magnetic field localization technology [21,22] could be adapted into compact, wearable, highly accurate proximity sensing system. The sensing modality is based on a low-frequency oscillating magnetic field, a robust concept that addresses the core challenge of reliability from RF-based social distancing sensing technique. We leverage the fact that the magnetic field strength that is generated by coils is attenuated at a cubic relationship with the distance: generating magnetic fields covering indoor scenarios, such as room, hallway, or factories, typically requires strong stationary sources with large coils having a high inductance. Smaller oscillating magnetic field systems suitable for wearable applications generate just enough radius for the range of social distancing, which means that unless large coils or high currents are used in order to generate strong magnetic fields, it is virtually impossible to sense any signal at more than around 2 m. While this has been a significant obstacle preventing using oscillating magnetic field systems for indoor localization, it makes the principle ideal for monitoring social distancing. This approach essentially creates a small magnetic field ‘bubble’ surrounding the moving user for their social distancing zone. Thus, we can detect social distancing violations without acquiring information about the user’s exact position.

To summarise, Table 1 lists the advantage and limitations of various approaches to social distance related proximity detection. The main limitation of our prototype is the current size of the hardware, which can be significantly reduced, as will be shown in Section 3.

As far as the authors know, we are the first to deploy this oscillating magnetic field-based modality for proximity sensing. The idea with a some initial results was summarized in [25]. In this paper, we provide a detailed description of the design, implementation, and evaluation of the system.

### 1.1. Contribution

Overall, we present the following contributions in this paper:

1. Description of the general concept based on the underlying physical principles.
2. Design of a system architecture including the analog circuits for signal processing, the coils and a protocol for decentralised coordination of multiple devices.
3. A wearable prototype systems optimized for highly reliable proximity detection around 2.0 m.
4. Characterization of the system properties in systematic, controlled lab experiments.
5. Evaluations in real-life city-scale scenarios showing near-perfect detection of the required social distance.

### 1.2. Paper Structure

In Section 1, we briefly introduce the motivation for our work. Section 2 examines the physical background and measurement principle. Section 3 describes the hardware implementation, especially the coils at both transmitter and receiver sides, as well as the core part of signal processing. Lab evaluation of the system’s range and robustness are stated in Section 4, followed by the real-life tests in Section 5, where two tests at different places, were performed, which demonstrated the the functionality of this novel proximity sensing modality for social distancing monitoring. Section 6 concludes our work and states future work.

## 2. Approach

### 2.1. Physical Background

A coil with a flowing current produces a magnetic field $\vec{B}\left(\vec{r}\right)$, where $\vec{r}$ is the distance from the center of the coil to the measured field point. The strength of the field $\vec{B}$ is proportional to the current *I* in the coil, and the direction of the field depends on $\vec{r}$.

For a more accurate approximation of the magnetic field layout, a finite element model using Maxwell’s electromagnetic theory can be explored, as described by Groben et al. [26]. Here, we describe a simplified situation where the measured point is along the normal of a coil. For a resonant coil, assume that the coordinate system’s origin is at the center of the coil and the *z* axis is the normal of the coil, then the magnetic field strength along the *z* axis is given in a simplified form by:

(1) $$\vec{B}=\frac{\mu_{0}na^{2}I}{2{\sqrt{\left(a^{2}+{\vec{z}}^{2}\right)}}^{3}}$$

where *B* is in Tesla, $\mu_{0}(4\pi *10^{-7}H/m)$ is the magnetic field permeability in the vacuum, *n* is the number of turns of the coil, *I* is the current in the wire, in Amperes, *a* is the radius of the coil in meters, and $\vec{z}$ is the measured point vector from the center of the coil. At a more considerable distance, the degradation of the magnetic field strength is inversely proportional to the cube of the distance.

Suppose that we have two coils, a transmitter coil with a sinusoidal current flowing (*w* is the angular frequency, φ is the phase of the current), and a receiver coil enclosed by a LC resonant circuit. According to Maxwell’s electromagnetic theory, a sinusoidal current flowing in the transmitter coil also generates a sinusoidally varying magnetic field around the coil (with the same AC characteristics in angular frequency and phase). Assume that $B_{p}$ is the peak-to-peak field intensity of a point in space, and then the magnitude of the magnetic field at that point generated by the transmitter coil can be written as:

(2) $$B=\frac{1}{2}B_{p}\cos\left(wt+\varphi \right)$$

In an oscillating magnetic field, according to Faraday’s law of induction, the voltage that is accumulated around a closed circuit is proportional to the time rate of change of the magnetic flux it encloses.

**Figure 1:**
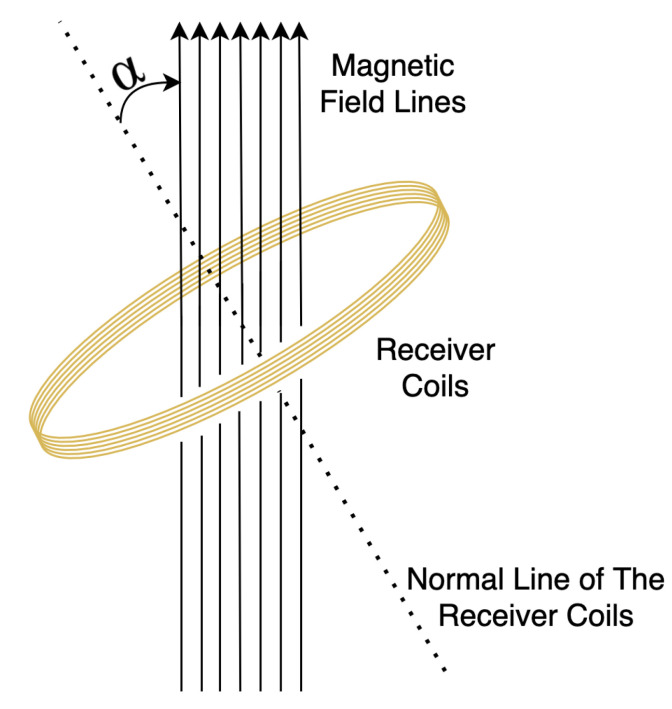
Receiver coil in an oscillating magnetic field.

As Figure 1 depicts, the magnetic flux through the receiver coil is:

(3) $$\Phi =\vec{B}\vec{S}=BS\cos\alpha$$

where *B* is the magnitude of the magnetic field (the magnetic flux density) having the unit of Wb/m${}^{2}$ (tesla), *S* is the area of the coil in m${}^{2}$, and α is the angle between the magnetic field lines and the normal to *S*. Assume that the coil has *n* turns, the induced voltage at the receiver coils will be:

(4) $$V=-n\frac{d\Phi }{dt}=-nS\cos\alpha \frac{dB}{dt}$$

Combining Equations (2) and (4), the voltage that is induced in the receive coil becomes

(5) $$V=\frac{1}{2}wnSB_{p}\cos\alpha \sin wt$$

When α equals to zero or π, the peak to peak value of the induced voltage can be written as:

(6) $$V_{p}=wnSB_{p}$$

### 2.2. Measurement Principle

From Equations (1) and (6), the induced peak to peak voltage at the receive coil is proportional to the angular frequency and the amplitude of the original current, and inversely proportional to the cube of the distance from transmitter coil to the measured position. Thus by sensing the voltage at the receiver side, distance information between the two coils can be deduced. The signal intensity will decrease rapidly as the distance gets larger; this rapid decrease has been one of the reason while oscillating magnetic field indoor positioning system have not become wide spread, since very large currents, very large coils, and most of all many transmitter coils would be needed in order to cover large rooms. However, for proximity detection related to social distancing requirements of around 2 m, this turns out to be perfect. With coils of a size (2–3 cm diameter) that is reasonable in a wearable system and typical currents (not more half ampere), the field is virtually undetectable outside a range of 2–3 m while being clearly present below those distances. Furthermore, the basic principle has two further advantages over radio frequency (and to a degree also ultrasound-based) based proximity sensing approaches:

1. The signal is NOT a propagating wave (such as RF or ultrasound signals) but a closed-loop field. Thus, we do not have to deal with refraction, reflection, and the resulting multi-path effects.
2. Magnetic fields are not significantly affected by objects in the environment. However, large ferromagnetic objects distort the field and are a problem when we want to address it in the future.

### 2.3. Transmitter Coordination and Identification

For the system to be useful for real life for social distance monitoring and exposure tracing, it must be able to function with many systems dynamically meeting each other. This requires synchronisation and device identification. Thus, the magnetic signal itself is not specific to any device. The signal is also “additive” means that, if two devices transmit at the same time, their signals would be summed up at the receiver creating the fingerprint of s single overlaid signal. To address the above problems, we use an RF (specifically Zigbee) “back channel” that we have implemented in two variations.

In small scale systems within constrained settings (e.g., a small workshop), we use a dedicated router that sequentially sends “transmit” commands to each sender. With the synchronization signal, all transmitters could be activated sequentially (50 ms activation time in a 70 ms time window for each), and all receivers have the same 166.7 Hz sample rate.

For large scale setting, we have implemented an asynchronous, scalable scheme similar to the ALOHA WiFi protocol. To this end, each system

1. Broadcasts its unique ID (Identifier) over RF (Zigbee or BT) at random time intervals.
2. Checks if there was a conflict during the broadcast (essentially another system broadcasting its message around the same time).
3. If there was a conflict then the systems waits a random period of time and goes back to step 1. Otherwise its proceeds to send the magnetic signal.
4. All systems that register a magnetic signal now know which sender it belongs to and can note to whom they have been in proximity.

For the above protocol to work, the RF synchronisation signal must have a larger range then the magnetic signal, which both Zigbee and BT by far have. On the other hand, the range should not exceed a few meters to avoid generating too many conflict, which again appropriate Zigbee modes and BT low energy fulfill (by adjusting their transmitting power). Note that the above identification scheme can use any ID generation and management method and is, thus, fully compatible with various privacy preserving approaches to contact tracing, such as the Apple/Google rolling identifiers decentralized exposure tracing method.

## 3. System Architecture and Implementation

### 3.1. System Architecture

**Figure 2:**
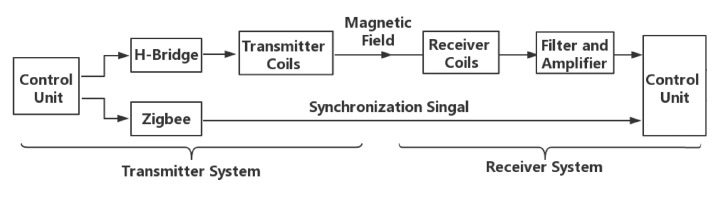
System Architecture.

Figure 2 illustrates the prototype’s hardware architecture. The system consists of two sub-systems, transmitter part and receiver part. The magnetic field transmitter continuously generates a magnetic field with a frequency of 20 kHz by driving an H-Bridge. At the receiver side, a receiver with coils in three axes measures the induced voltage, which is then filtered, amplified, and digitalized. For evaluation, the data are stored locally into the SD card. The size of the prototype is 16 cm × 6.5 cm × 6 cm. Our prototype’s size is restricted by the modular boards and size of the rechargeable battery and can be optimized and integrated into smaller footprints in the future.

**Figure 3:**
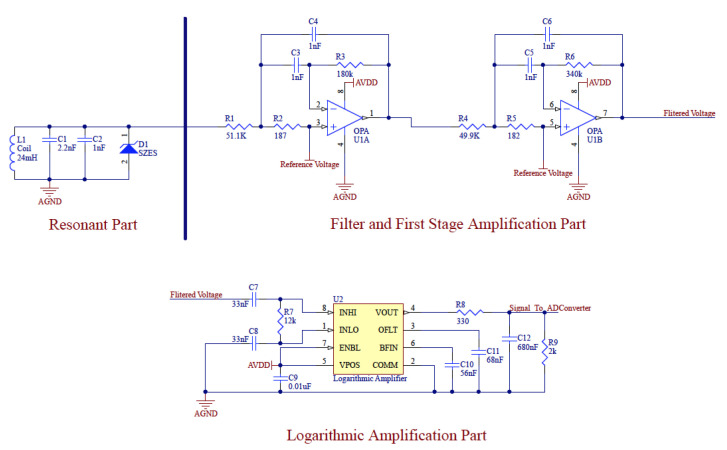
Schematic of the Butterworth filter and logarithmic amplification chain.

**Figure 4:**
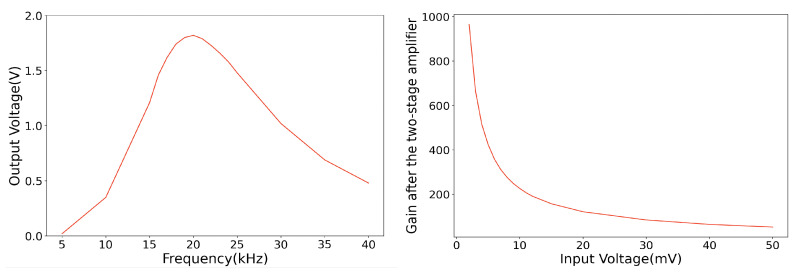
The filter response of the Butterworth filter (input voltage: 20 mV) and the signal gain after the Butter-worth filter and the logarithmic amplification.

Figure 3 shows the signal processing circuit at the receiver side. Firstly, it filters and amplifies the voltage induced by the oscillating magnetic field by a four-order Butter-worth filter to provide a maximally-flat response and high roll-off (40 dB/dec). Thus, the input signal for the next stage is stable and of low noise (Figure 4 left). An impedance matched logarithmic amplifier is then connected to the filter, aiming to compress the preamp output signal with a wide dynamic range to its decibel equivalent via a precise nonlinear transformation (Figure 4 right). Finally, the signal was sampled by a 24-bits resolution analog to digital converter.

The receiver system consumes 120 mA current with a 3.7 V power supply. The transmitter system consumes 170 mA current with a 10 V power supply. A rechargeable 1500 mAH battery supplies the power with an 11.1 V output and supports a working time of more than five hours.

### 3.2. Transmitter and Receiver Coils

**Figure 5:**
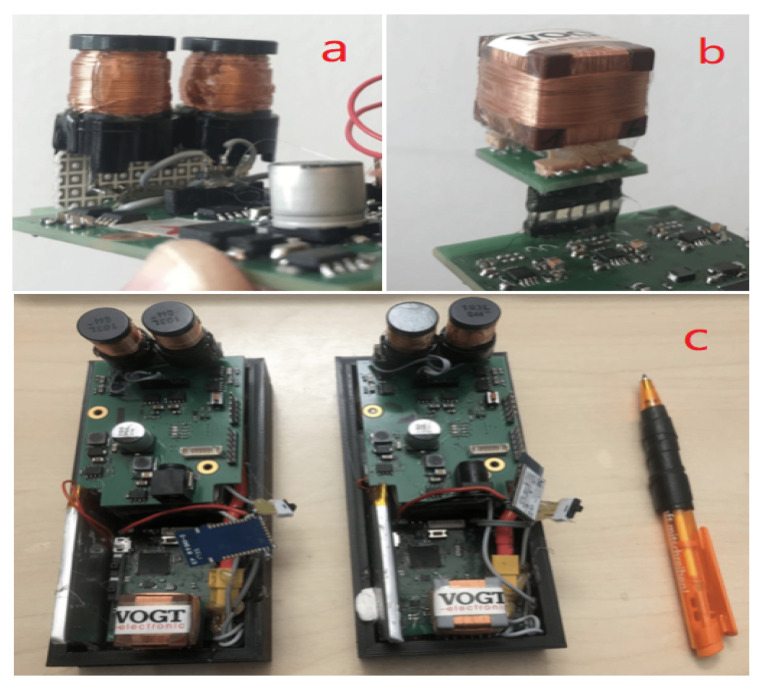
Transmitter coil (**a**), Receiver coil (**b**), and two prototypes (**c**).

The reliability of our proximity evaluation system depends on the measurement accuracy of the generated magnetic field strength. The transmitter coils (Figure 5a are two serially connected commercial inductors with an inductance of 3.3 mH for each. The low resistance of the coil benefits a high resonant voltage after tunning to the resonant circuit. The height of the inductor is around 20 mm. The coil at the receiver side (Figure 5b with an inductance of 10 mH is a customized one with the material of MnZn-ferrite, enjoying low losses and high saturation induction. Three sets of coil enclose a cube structure, being able to sense the magnetic field in the three perpendicular directions. Since the coils overlap at each side, the capacitive crosstalk is thus generated. The tests showed that this crosstalk could positively increase the receiver system’s sensitivity instead of introducing the noise. The length of the cubic coil is around 18 mm. The transmitter coil and receiver coil are both tuned to a resonant circuit with a resonant frequency of 20 KHz.

While choosing the best coils for our prototype, the Q-Factor is one of the most critical parameters for consideration, describing the ‘sharpness’ of a resonant circuit. It is calculated by dividing the resonant frequency by the bandwidth. The higher the Q-Factor, the smaller the bandwidth, thus the sharper the resonant voltage’s frequency response. A higher Q-Factor also means a better frequency selectivity of the circuit, since any frequency outside the bandwidth will be quickly rejected. To summarize, at the transmitter side, a higher Q-Factor means less energy loss of the circuit; at the receiver side, a higher Q-Factor means a better selectivity for the oscillating signal.

### 3.3. Minimization Potential

**Figure 6:**
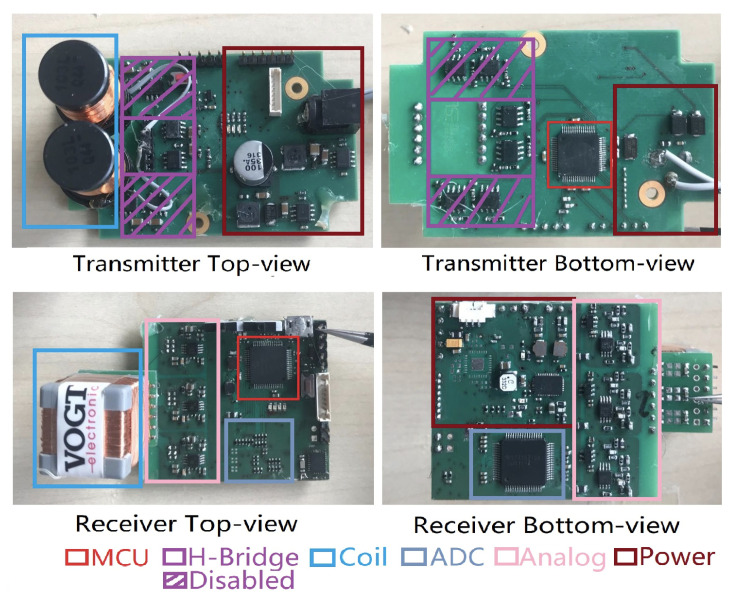
Minimization potential of the prototype Printed circuit board (PCB), where firstly, a plenty of free space exists, especially on the transmitter board; secondly, there is significant redundancy in terms of modules on both transmitter and receiver boards, for example, the micro-controller module and the power module.

The prototype depicted in Figure 5c was built as a “rapid prototype” that needed to be small enough to permit real-world experiments, but with no emphasis on miniaturization. In fact, it was, to a large part, built on components adapted from our previous indoor location system [21], in which the size and power consumption of the hardware system was not considered at all. Consequently, the system has significant potential for further miniaturization:

1. Coils. The current transmitter coil is a cylinder-shaped inductor with a 1.6 centimeters radius and 2.0 cm height. The receiver cubic coil has a 10 mH inductance in each axis. Both coils could be optimized in size with further customization while maintaining the inductivity and sensitivity. However, reducing the coil size implies reducing signal strength, and making a more sensitive receiver or higher currents necessary. This means that, with the current system concept, a coils size of much less then 1 cm is unlikely. However, we could use analog multiplexers to share a single coil between the receiver and the transmitter circuit, reducing the space needed for the coils by half.
2. Battery. The current prototype uses a battery commonly found in drones as it can easily provide the voltage (10 V) and currents needed by our prototype. However, with some optimization, a much smaller, standard smartphone, or even smartwatch battery could be used. First of all, with a voltage boost converter, we could make the use of a standard 3.7 V battery pack. Second, by extensive use of a sleep mode for the magnetic receiver and transmitter, up to 90% of the required power could be saved. The transmitter and receiver operate continuously, drawing 170 mA (at 10 V) and 120 mA (at 3.7 V) respectively, with a total of around 2 W power consumption, as described in Section 3.1. However, in reality, each system needs to transmit a magnetic signal for 50 ms ever few seconds, which amounts to a duty cycle of less than 5%. Depending on the number of other people in the vicinity, the receiver will need a higher duty cycle. However, because we are looking at the proximity within a 2 m radius in general, we are unlikely to see more than a few people, so that a duty cycle of 10–20% is also realistic. The magnetic components’ duty cycling will be driven by RF synchronization signals that would need to leave enough time between the RF signal and the following magnetic signal to allow all of the receiver to be switched on and initialized. The power consumption could also be optimized further with low power design. As an example of design towards low power, the micro-controller can be replaced to nRF52 series [27] from the Nordic Semiconductor, which consumes power with a level of mA while using the on-chip integrated wireless module and with a level of uA while in sleep mode. In summary, the power consumption could easily be reduced by a factor of around 10 to be somewhere in the range of 200 mW even less with an ASCI (Application Specific Integrated Circuit) design [28,29], as discussed below. For 5–6 h of operation, a battery of 1 Wh (less than 300 mAh at 3.5 V) would be needed, which corresponds to the battery packs currently used for many smartwatches (296 mAh for Apple Watch 5, for example).
3. PCB (Printed circuit board) size. The current transmitter board is a two-layer PCB with a low component density, as shown in Figure 6. Besides, there is significant redundancy in terms of components between the transmitter and receiver boards. As a first step, we could use only one power module and one microcontroller. Secondly, because we only use one axis for magnetic field generation at the transmitter side, only one channel of the H-Bridge module is needed. Finally, many of the components are available in much smaller footprint packages. As an example, the footprint for the amplifiers in the analog module could be changed from the current TSSOP-8 footprint to a much smaller SOT-23 footprint; other high-resolution ADCs could replace the current ADC chip (ADS1298) with a smaller package. Besides the components, the onboard pins and wires on the prototype can be grouped into only four modules, the power module (voltage and ground), the microcontroller module, the H-Bridge module, and the analog circuit module (three operational amplifiers and the surrounding passive components), which means the density of connections (pins and wires) will not hinder minimization. Moreover, all of those pins and wires can be laid out quickly on a PCB with more layers [30,31] to further increase the density.

**Figure 7:**
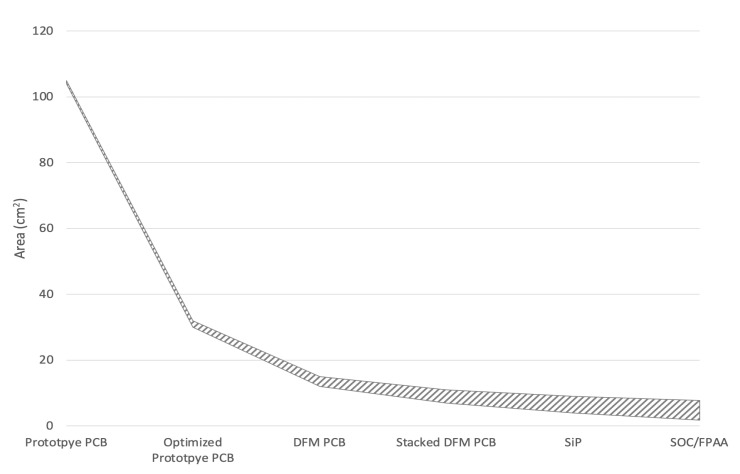
Board/Package Area Projection with Different Levels of Design Integration, starting from the current prototype with the PCB size of 104 cm${}^{2}$, followed by an optimised PCB with a 20% components density and a design for manufacturability (DFM) design with a 50% components density; further minimisation could be performed by a stacked PCB or SiP (system in package) design, or by the chip-level FPAA/SoC design.

We estimate that with some basic optimization techniques and high end PCB technology a devices size in the range of 5.4 cm × 3.5 cm × 4.1 cm (coils included) is feasible, as shown in Table 2. Such a device could easily be carried in a pocket, on a belt clip, etc. The further reduction would be possible with advanced electronic packaging techniques. For example, because the analog components required in our prototype are discrete surface-mount components requiring only three amplifiers each channel with the surrounding passive components, we can consider integrating the analog channels into a single system-on-chip die or possibly FPAA (field-programmable analog arrays) [32,33] for further miniaturization, leading the PCB size to that similar as a smartwatch on the market. The H-Bridge is nothing more than four MOS transistors, which can be integrated into a controller chip with power management functionality, the so-called Motor-Driver-Controller [34,35], like the STSPIN32F0A from STMicroelectronics as an example of the SoC (system on chip) design. Figure 7 contains a summary and visualization of the above analysis.

To summarize, even with some relatively straight forward optimizations, such as duty cycling the magnetic signaling, using small-footprint components, and high-end PCB layout, the system size can be reduced to a “stack of credit cards” form and can easily be carried in a pocket. Looking towards mass deployment, advanced packaging and SoC techniques would allow for a smartwatch-like form to be easily worn, e.g., on a wrist.

## 4. Lab Evaluation

We first performed the sensing range and robustness tests in labor to evaluate this magnetic field-based system’s proximity sensing ability.

### 4.1. Sensing Range Test

**Figure 8:**
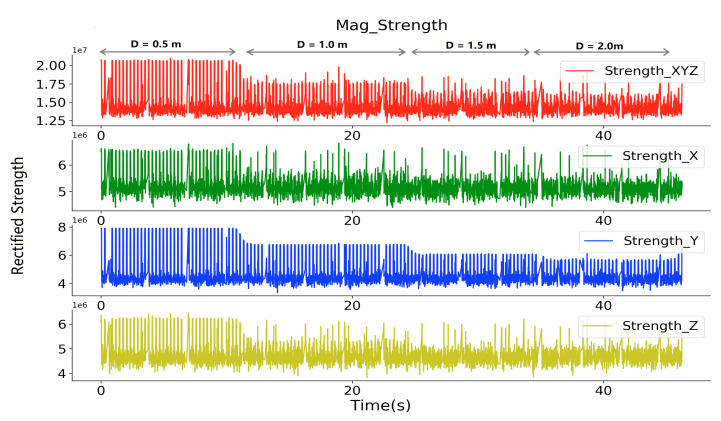
Sampled magnetic field rectified strength in the detection range test with one transmitter activated.

We moved a receiver away from a transmitter in a controlled tabletop experiment to establish a baseline for our implementation. Figure 8 shows the perceived magnetic field strength information with different distances between an activated transmitter and the sampling receiver. The transmitter system of one prototype was activated and fixed on a table, the receiver system of another prototype sampled the magnetic field strength in three-axis at different spots in range of 0.5 to 2.0 m. The depicted strength *Y* shows a detection range of beyond 2.0 m.

The data from the first subplot were calculated by the root sum squared of the data sampled by the three coils at the receiver side, and showed a less clear strength variation along with the distance variation as compared with the axis of *Y*. Which is reasonable, since the other two axis’s data at a broader range is mostly noise. Thus, we decided to use the raw data sampled from the *Y* axis as the source information to deduce the distance. This means that we either have to mount all of the devices in the same orientation on the body (which we did for our prototypes) or have a transmitter generating magnetic field on all three axes (which is essentially just increasing circuit complexity, which we wanted to avoid, but have done for our magnetic indoor positioning [21]).

To be noticed, the Strength depicted in this figure, as well as the following figures, is not the practical magnetic field strength with the unit of Tesla. It is the raw value from the analog to digital converter, representing the strength in a way. Because the practical strength value is not easy to be back-deduced after the signal is being processed by the analog signal processing circuit (Figure 3), especially being non-linearly amplified by the logarithmic amplifier. Another reason that we use this raw value to interpret the magnetic field strength is that our interest locates only in the distance information, which can be deduced by a fitting method with the sampled raw data.

**Figure 9:**
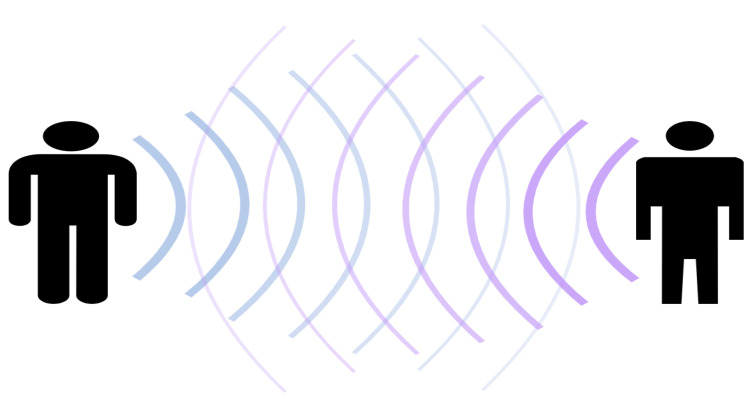
People in the range of the magnetic field while wearing the prototype on bodies.

**Figure 10:**
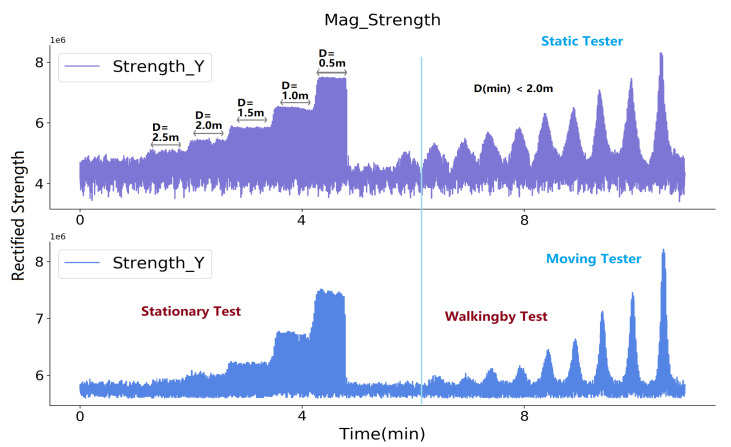
Detection range test with two body-worn prototypes activated. One in stationary state and another in mobile state.

**Figure 11:**
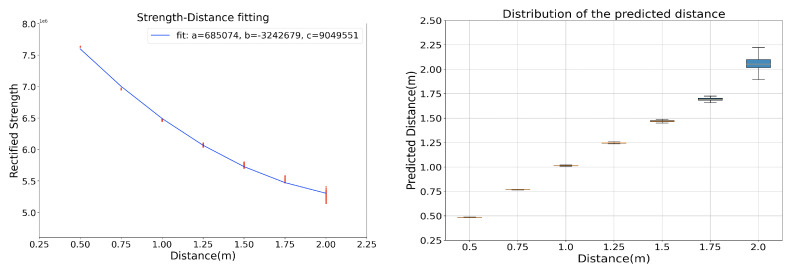
Fitted strength-distance relation and the distribution of the predicted distance with the fitting equation with a Boxplot.

A second detection range test was performed by deploying the prototypes on the bodies. A case was printed to hold the hardware system and it was tied to the body by a flexible band. Two volunteers took part in the test in the labor place. Once they were close to each other, the magnetic system would be able to sense another magnetic field, as Figure 9 depicts. Figure 10 shows the sampled strength signal. Firstly, P1 and P2 both stood statically with different distances between them (from 3.5 m to 0.5 m). Subsequently, P1 switched off both transmitters by its router and walked away. Then switched on to activate both transmitters and performed walking by events again with different minimum distances. The signals from both receivers demonstrated that both prototypes have a detection range of beyond 2.0 m; secondly, they might have various sensitivities and offset levels that are caused by the hardware drivers, meaning an individual calibration is needed. Because all of the drivers were manually soldered and debugged, constant sensitivities, noise levels, offset levels can not be guaranteed. Thus, to deduce the distance information, we used a curve fitting method to summarise the strength-distance relationship for each prototype. The left of Figure 11 exemplifies one of the prototype’s strength-distance relationship by a quadratic equation with the corresponding parameters. To get the strength-distance equation parameters, we sampled 500 pairwise strength-distance values for seven distances (0.5 m to 2.0 m, with an interval of 0.25 m) and made a curve-fitting. Figure 11 right uses Boxplot [39] to summary the frequency distribution of the estimated distance from the rectified strength signal, where the first quartile, median, and third quartile of the predicted value are depicted. The Boxplot shows that the predicted distance values mostly distributes in the range of 0.1 m of the actual distance and the distribution range becomes smaller when the actual distance decreases. Table 3 lists the mean and standard deviation of the errors after distance estimation. When the range is within 1.5 m, the estimation error’s mean is below 3 cm. The error’s mean will increase while the actual distance goes beyond 1.5 m, but still less than 10 cm, which means that this curve-fitting distance estimation approach from the rectified strength value is acceptable. The other prototypes have a similar strength-distance relationship, but the parameters are slightly different. The parameter *a* mainly relates to the gradient of the quadratic relationship, and parameter *c* mainly influenced by the offset level.

### 4.2. Robustness Test

The detection range test result gave us a functionality-successful view of our magnetic field-based proximity sensing modality. In this part, we demonstrated the robustness of our system concerning disturbance through different objects in the environment, which belongs to the main advantage features over the other proximity sensing modalities.

**Figure 12:**
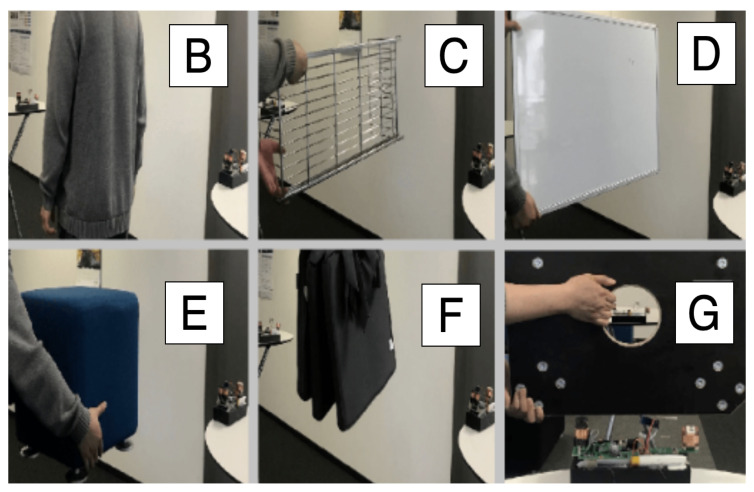
Robustness test with various obstacles between two prototypes: in case (**B**), a human body as the obstacle; case (**C**–**G**) with obstacles of a metal frame, plastic, sofa with wood and metal, thick textile, and thick wood, respectively. The corresponding rectified strength signal is depicted in Figure 14.

We put two prototypes on two tables with a fixed 1.5 m distance, different obstacles from Figure 12 were placed once at a time between the two prototypes. The transmitters were deactivated while switching the objects.

**Figure 13:**
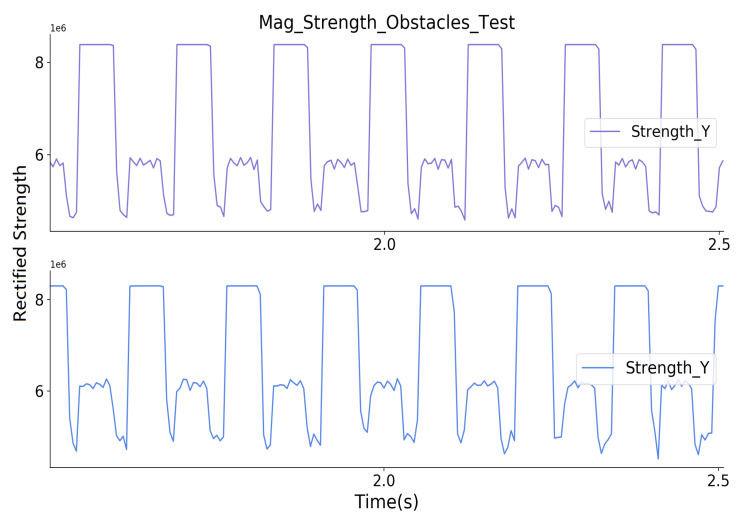
Close look of the received strength signals from both prototypes.

Figure 13 shows a close look at the received magnetic field strength of both receivers. Both of the transmitters are activated for 50 ms in a 70 ms window. The prototype’s transmitter itself undoubtedly generates the wide peaks with bigger values seen by the prototype’s receiver, the wide peaks with smaller values are the sampled magnetic field strength generated by another prototype. Thus, the robustness demonstration fails if this value varies with different obstacles between the prototypes.

**Figure 14:**
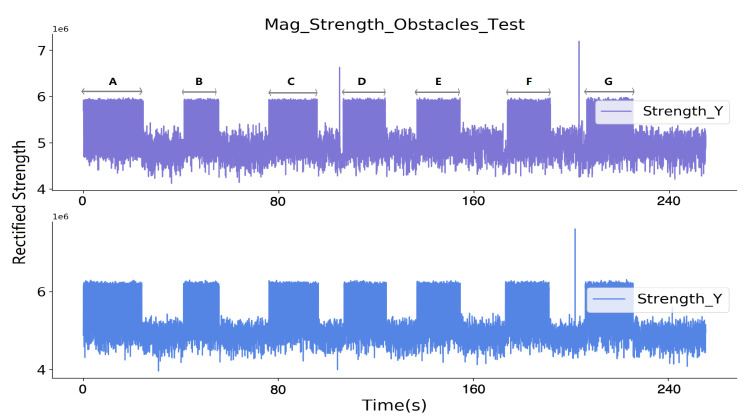
Received strength signals from two prototypes after removing prototype’s own magnetic field, where nothing in between in time window A and different obstacles in the other six time windows.

Figure 14 shows the perceived strength of each prototype after removing the strength generated by their own transmitter. With period A having nothing between the systems and the other periods with the six everyday objects in between, the registered signal strength was not altered by those obstacles, which demonstrated the robustness of our proximity sensing modality.

### 4.3. Randomly Walking-By

**Figure 15:**
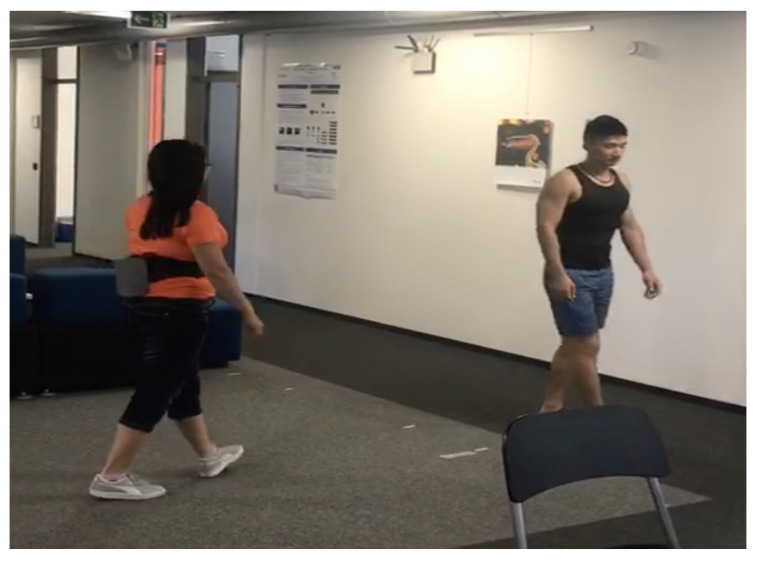
Two volunteers with the prototype on body performing randomly walking-by events without any instructions at an empty area with size of 5 × 5 m.

**Figure 16:**
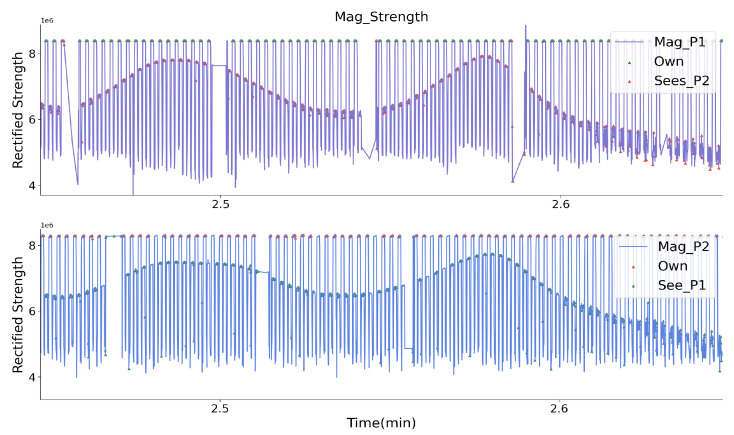
A close look of TDMA (Time-division multiple access) based strength signal from two prototypes during a randomly walking-by, the peaks with constant amplitude indicates the field strength from its’ own transmitter, the peaks with a varying amplitude indicates the field strength from another prototype, which indicates the actual distance.

**Figure 17:**
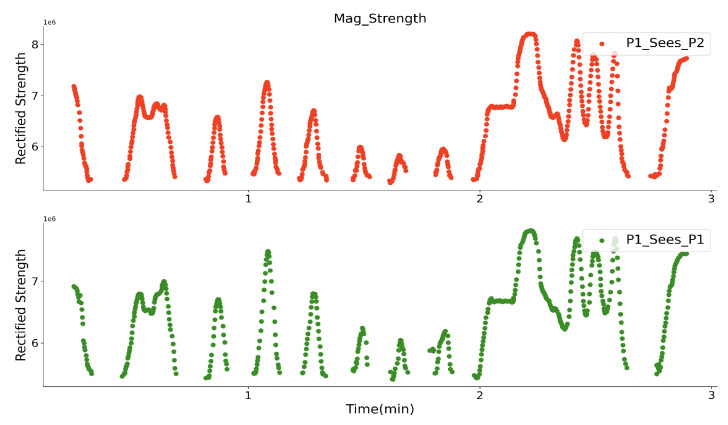
How each volunteer sees the walker-by in strength.

**Figure 18:**
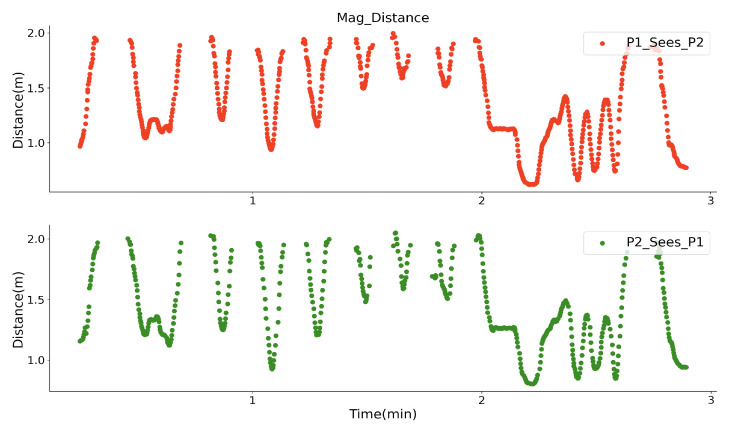
How each volunteer sees the walker-by in distance.

As a final stage of the lab evaluation, two volunteers wore the prototypes, as depicted in Figure 15, and performed a randomly walking-by test in three minutes without any instructions in an empty area with a size of 5 × 5 m. They wore both the prototypes on the back with a flexible band. Both of the prototypes had the same direction and orientation in space, aiming to ensure that the *Y* axis of the receiver coils always gets the strongest magnetic field, and the sampled strength of the two prototypes is comparable with each other. They walked in any directions, so the walking-by events happened randomly. During the test, they stood statically with a shorter range for a short time. One prototype’s Zigbee worked as the server broadcasting the current activated prototype ID with a 70 ms period. Once the prototype obtains its ID, its transmitter system will be activated for 50 ms. Figure 16 depicts a closer look of the sampled strength signal for each prototype. The strength with higher peaks represents the signal from its own transmitter part, since the nearest transmitter is the one from the same prototype, and they have the constant distance, thus the sampled strength keeps the same value. The other peaks with varying amplitudes are strength information from another prototype. As the trajectory of the peaks shows, the strength of the other prototype generated magnetic field is varying, since the distance between the prototypes were changing. After removing prototype’s own magnetic field signal and setting a threshold for an adequate strength level, as Figure 17 shows, each volunteer could ‘see’ another one, while they were in a specific range. The strength of the signal represents the distance between them. After the second minute, the strength sampled by both prototype keeps unchanged for a while, this means that the two volunteers were standing statically for that period. By applying the strength with the quadratic relationship (Figure 11), we get the practical distance information between them, as Figure 18 shows. The interpreted distance information clearly shows the walking actions of the two volunteers. For example, in the first two minutes they walked by for seven times (besides the beginning where they stood close to each other), and nearest one happened at around the 65th seconds with a distance of below one meter. They stood statically for a while after the second minute with a distance of around 1.1 m. With the prototype, we successfully recorded the social distancing for each volunteer during this randomly walking-by tests. Although we have no ground-truth distance data during the test, the similarity of “P1_sees_P2” and “P2_sees_P1” could demonstrate the truth of distance between them in another way, which means that our magnetic field-based proximity sensing prototype can efficiently perceive the distance information in a range of 2 m and that the usage of the quadratic relationship (strength to distance) from a curve fitting method is acceptable.

## 5. Real World Experiment

To demonstrate the feasibility of our approach in practical scenarios, we performed two tests: a 16 min. group walking in a construction material supermarket with Zigbee-based server for synchronization and a 100 min. city-scale group walking with an asynchronous protocol.

### 5.1. Supermarket Walking

In the previous lab experiment, we tested whether different objects in everyday life could influence the distribution of the magnetic field and, thus, blocking the social distancing monitoring with our prototype. The test showed a positive result. In this subsection, we put our prototypes in a more complex and dynamic environment, a construction material supermarket, where plenty of goods made of metal, plastic, wood, cement, exist, and other individuals were wandering in the market. Four volunteers worn the prototypes and walked outside and inside the supermarket. They all wore face masks during the test.

**Figure 19:**
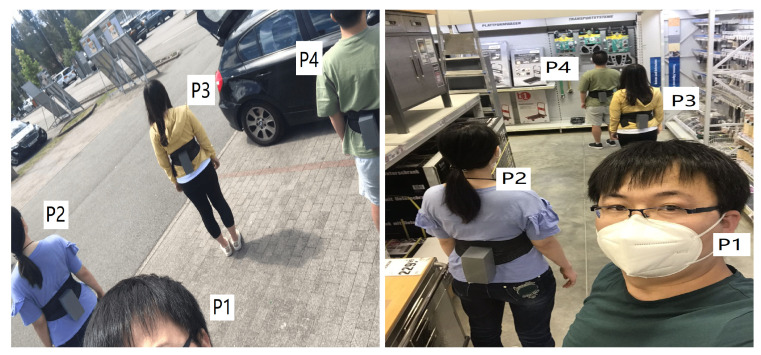
Four volunteers’ practical test outside and inside the construction material supermarket where a more complex environment exists. P1 and P2 as one group with a distance of 1.0 m and P3 and P4 as another group with a distance of around 1.5 meters, the distance between the two groups is beyond 3.0 m.

As Figure 19 shows, at the beginning of the evaluation, four volunteers stand close to each other for a while outside the supermarket within range of two meters. Then two of them (P1 and P2) as one group kept a close distance of around 1 m and the other two (P3 and P4) as another group with a distance of around 1.5 m (two arms’ length). The distance between these two groups was kept beyond 3 m. They wandered in the supermarket for around 14 min. and then exited the market to the starting place. Again, at the starting place, the two groups kept a closer distance.

**Figure 20:**
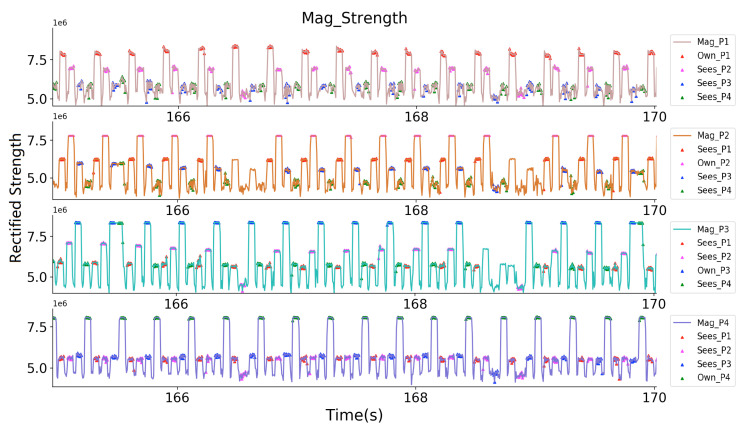
Four Volunteers’ receiver sampled rectified magnetic field strength signal, where they can see each other.

**Figure 21:**
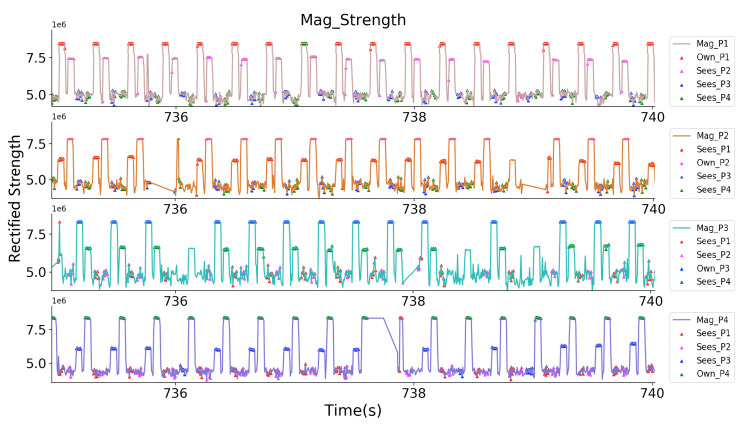
Four Volunteers’ receiver sampled rectified magnetic field strength signal, where they can see only another volunteer in their group.

Again, here we used one of the prototype’s Zigbee to synchronize all of the transmitters and receivers. Figure 20 and Figure 21 give a closer look at the receiver sampled raw strength signal at the two 5 s time windows while the volunteers were at the starting place and inside in the supermarket. Figure 20 depicts how each volunteer ‘sees’ the others. For example, in the subplot of P3, where his own magnetic field gave the strongest strength, and the other three magnetic fields gave weaker sampled strength, but can still be sensed. Among the three fields, the fields from P2 shows the strongest strength, meaning P2 has the nearest range to P3. Figure 21 shows that the volunteers can only ‘see’ another volunteer in their group (for example, P1 only sensed P2’s magnetic field, and P3 only sensed P4’s magnetic field). The prototype can not sense the magnetic field generated by another group’s prototypes, since they were beyond the range of three meters, although the identifiers of the prototypes were successfully received.

**Figure 22:**
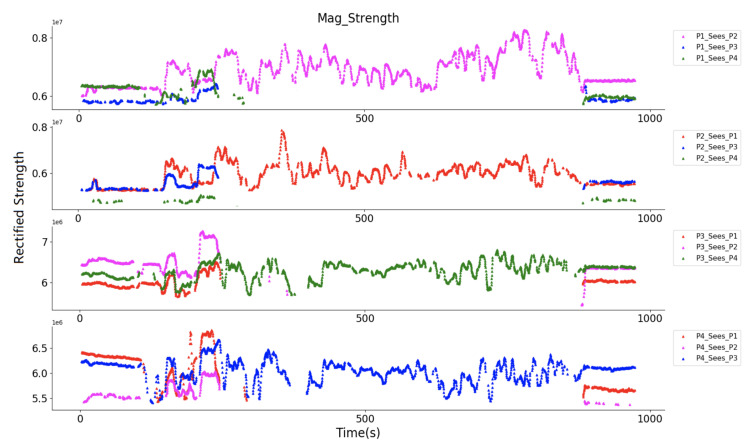
Perceived magnetic field strength of each prototype in the whole test.

**Figure 23:**
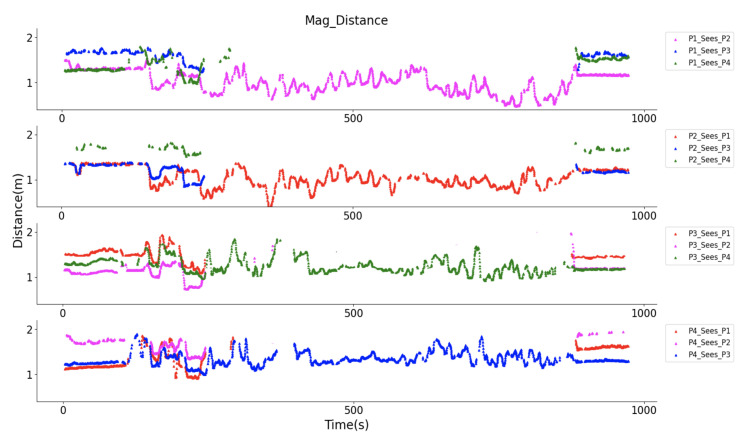
Distance tracking of four volunteers in a construction material supermarket, where at the beginning and end they gathered together, and during the mid-time they kept a group distance of beyond 3 m.

Figure 22 gives an overview of the observed magnetic field strength for each volunteer after the strength data was smoothed by a moving average method with a 10 s window. A stronger rectified strength means a closer distance between to volunteers. The field strength variation during the whole walking was obviously represented. The bias of each signal strength in Figure 22 is not identical, which is reasonable, since the hardware cannot supply the same reference voltage in the accuracy level of mv (Figure 3), a 10 mV difference in reference voltage will cause 1.6 × 10^6^ bias difference in the rectified strength. At the starting and ending period, all the other volunteers could be “seen”. During the middle period, only one volunteer was within the detection range. After interpreting the strength information into the practical distance with the quadratic equation, the social distance of each volunteer is depicted in Figure 23. In P4’ view, at the beginning, P1 and P3 were standing with a distance of around 1.2 m, and P2 with a distance of around 1.8 m, that distance information was also reflected in the view of P1, P2, and P3 as the same values. During the mid-time, P3 and P4 kept a distance of around 1.5 m, P1 and P2 kept a distance of below one meter, which matches the actual configuration during the test. Besides the distance information, again here we see the similarity of “P1_sees_P2” and “P2_sees_P1” (or “P3_sees_P4” and “P4_sees_P3”), which demonstrates the truth of distance between them in another way. This practical test of the magnetic field based proximity sensing in the construction material supermarket could demonstrate its robustness and reliability for social distancing monitoring.

### 5.2. City-Scale Walking

We removed the synchronization scheme and applied an asynchronous protocol for the prototypes to ensure the mass deployment of the prototypes. Each transmitter system broadcasts its ID with a random length of period and then activates a magnetic field. An LFSR (linear-feedback shift register) based pseudo-random number generator [40] on the chip supplies the period value, which ranges from 1 s to 2.55 s. With the asynchronous protocol, four volunteers wore the prototypes and walked from our working building to four different supermarket, including one media supermarket, one construction material supermarket, and two general supermarkets, then walked back. The test lasted around 100 min with more than 8 km route. Again, we separated the volunteers into two groups: P1 and P2 were one group walked side by side, P3 and P4 as another group walked with a distance of around 1.5 m. The two groups kept a distance beyond 5 m.

**Figure 24:**
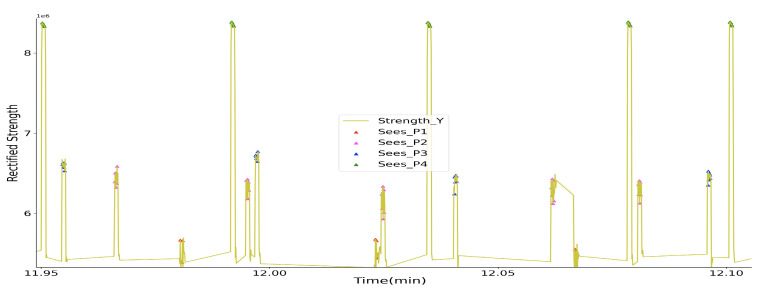
A close look of the asynchronous protocol, where each transmitter being activated after a random length of time window, and the receiver firstly listens for an identifier, then samples the strength after an identifier is received.

Figure 24 shows the sampled rectified strength data from P4’s prototype, where each transmitter was activated after a time window with a random period. Once the random time runs over, the transmitter will firstly broadcast its identifier, and then activate the magnetic field for 50 ms. The receivers firstly listen to the identifier information and then sample the field strength for the same time once an identifier is received. As the figure depicts, P4’s transmitter gives the strongest field strength and activates the field with an irregular period, the other three magnetic fields are also activated and sampled with a random period. The adoption of asynchronous protocol enables the mass deployment of our prototypes; meanwhile, an important point needs to be noticed, namely the random length of the time windows, which will affect the number of prototypes in the deployment. We currently give 1 s to 2.55 s interval to the windows and 50 ms for each of the field activation. If a longer interval is set, then some social contact will be missed. If a shorter interval is set, there will be more conflict from the transmitters where an overlapped magnetic field occurs.

**Figure 25:**
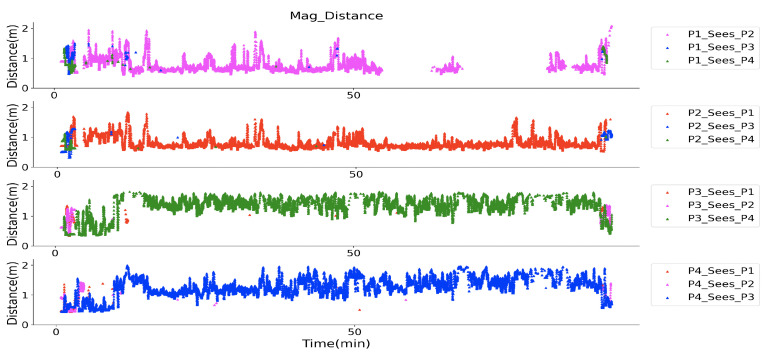
Distance deduced by our prototype during the city-walk where P1 and P2 as one group with a distance of 1.0 m and P3 and P4 as another group with a distance of around 1.5 m, and the distance between the two groups is beyond 3.0 m.

Figure 25 gives the distance information during the whole walk, where P1 and P2 kept a range of less than one meter most of the time, P3 and P4’s distance was mostly around two arms’ length. Both distance information matches the practical situation. At the very beginning and end, they were close to each other, which was also reflected in the strength interpreted distance recording. Some break of distance in group was also captured, for example, at the 14–15 min., P1 and P2 had a larger distance than the instructed one meter. Because the walk lasted for a long time, the in-group distance was not kept strictly and varied in a small range, especially the distance between P3 and P4, where the distance frequently varied between one and two meters. The high distance similarity of P1 ‘sees’ P2 and P2 ‘sees’ P1 during the whole walk also shows the reliability of the prototypes. However, this city-scale experiment uncovered some hidden flaws of the system. Firstly, a mechanically stable prototype is necessary, aiming to prevent waggle caused failure during long time use cases, as the gaps shown in the first subplot of Figure 25. This can be addressed by integrating the transmitter and receiver systems with a smaller circuit board, removing unnecessary flat cables. Secondly, although a very high accuracy of distance monitoring is not a necessity for social distancing, we tend to supply that information as accurate as possible, and there is still space for accuracy improvement of our prototype (as the third and fourth subplots in Figure 25 at around 15 to 20 min. depicted, there was a noticeable distance difference of “P3 sees P4” and “P4 sees P3”). The reason could be the larger windows size between two field activation, could be the curve-fitting bias during the distance information abstraction, or the mechanical orientation shift of the receiver. To provide a stable and high-accuracy system will be one of our future works.

In addition to the magnetic field-based proximity sensing prototypes, we ran a simple Bluetooth scanner application during the city-walk, where each of our iPhones broadcast a legible ID (to circumvent the Bluetooth MAC anonymization strategy [41]), while at the same time scanning for signals from the other iPhones (based on their known IDs). This was to illustrate how our system compares to established contact tracking [11,12,42] approaches.

**Figure 26:**
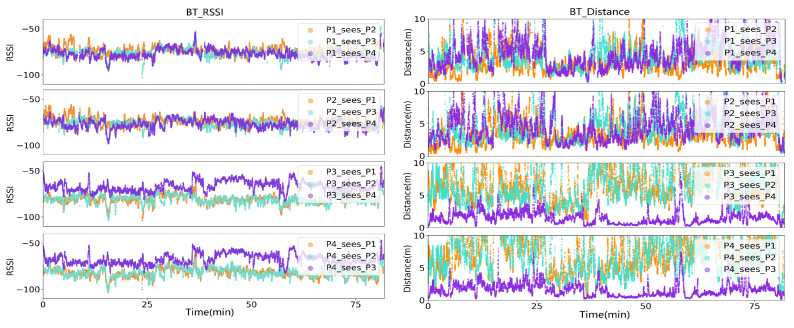
Bluetooth (BT) based Received Signal Strength Indicator (RSSI)/Distance information during the city-walk.

Figure 26 (left) shows the smoothed Bluetooth RSSI (Received Signal Strength Indicator) signals supplied by smartphones, where each volunteer hold a smartphone, sampling the near field Bluetooth strength, and meanwhile being sampled by other smartphone. The difference of the signal strength is not nearly as pronounced as in the case of the proposed magnetic field sensor. While for P3/P4, the signal from his immediate partner walking next to him (P4/P3) is consistently stronger than any other signal (as would be expected and desirable), this is neither the case for P1 or P2. As the first subplot depicts, at P1’s view, P2, P3, P4 had nearly the same level RSSI signals, although P3/P4 were much further than P2.

**Figure 27:**
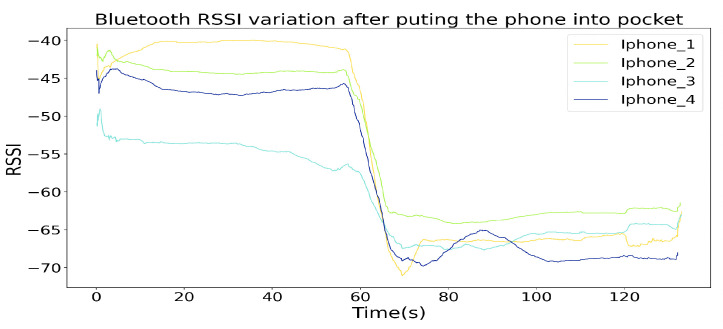
RSSI changed significantly after putting the BT RSSI sampling Iphone into the pocket.

Figure 26 (right) was deduced by applying the most commonly used RSSI-to-Distance theoretical model [43,44]:

(7) $$d=d_{0}10^{\frac{RSSI_{d_{0}}-RSSI}{10n}}$$

where *d* is the distance between the place of RSSI measurement and the equipment, $RSSI_{d_{0}}$ is the RSSI value measured from distance of $d_{0}$, RSSI is the value currently measured, and *n* is a constant depending on the Environmental factor, normally with range of 2–4. The estimated distance by Bluetooth RSSI value shows an unreliable estimation. Firstly, in P1’s view, P3 and P4 in the middle period can not show a closer distance than P2. Secondly, the estimation of the distance between P1 and P2 (or P3 and P4) should not go beyond 2 m during the whole walk. This means that the Bluetooth RSSI based social distancing monitoring tool is not as accurate and robust as the magnetic field-based approach. The reason behind the unreliability of Bluetooth RSSI could be firstly the environment variables. Secondly, the smartphone was holding in different ways. For example, as Figure 27 shows, after putting the BT RSSI sampling Iphone in the pocket, the RSSI signals from the other four BT RSSI broadcasting Iphones significantly attenuates, although the practical distances between the signal sampling iPhone and signal broadcasting iPhones did not change a lot. To be noticed, the methods for RSSI to distance transformation that we used here is the most theoretical one, by adopting compressive sensing principle [45,46], the result of BT-based social distancing estimation could be improved. Some other researches also showed that BT-based RSSI for distance estimation is acceptable in fuzzy classes (like close, near, far), but not in accurate distance calculation [47,48].

## 6. Conclusions

The results that are presented in this paper show that oscillating magnetic field systems can very reliably detect proximity relevant for COVID-19 social distancing and exposure tracing. Thanks to the $d^{3}$ dependence of the signal strength on the distance with a coil size of around 1–2 cm and current of less than a half ampere, we obtain a signal that virtually disappears outside the social distancing range of 2–3 m while providing good distance estimation for smaller ranges. The detection quality is further enhanced by the fact that our approach has no problems with multipath propagation and refraction, which are common issues with RF or ultrasound-based systems. This is due to the fact that we work with expanding and retracting the field, which never leaves the source and propagates (as do RF signals). One of the core challenge for our prototype locates at the identifier management, aiming to overcome the privacy concern. With the asynchronous coordination and transmitter identification protocol, the concept can be deployed on a large scale and it is compatible with current privacy-preserving exposure tracking systems, such as the Apple/Google protocol used by many “Corona Apps” around the world. In fact, the system could easily be used to extend such Apps in settings where more reliable exact tracing is needed or where difficult environmental conditions hamper the BT proximity detection accuracy. To this end, we would essentially only need to (1) replace our own RF-ID broadcasts with the Bluetooth ID broadcasts that such Apps do anyway and (2) include the magnetic proximity information in the risk assessment computation of the App. Thus, the user would be able to rely on the standard App when the magnetic sensor is not available while making sure that the use of magnetic information contributes to the overall exposure sensing process. Another challenge is the portability of our prototype, as discussed in previous section, there is a huge space for prototype improvement related to size minimisation and low power design. After the investigation of such integration with standard contact tracing Apps and improvement in portability, this magnetic field based proximity sensing device could be perfectly used for social distancing during the pandemic.

## Figures and Tables

**Table 1:** Potential proximity sensing technique for social distancing.

Approaches	Advantages	Limitations
Ultra-sound [14]	high accuracy, large proximity range	low robustness, environment dependency
Ultra-wideband [23]	high accuracy/portability, large proximity range	high cost, base station requirement
Sound-Fingerprint [24]	ease-of-use(smartphone)	environment dependency, sound exchange
Capacitive body sensing [15]	low cost/power-consumption	environment dependency, short proximity range(below 1.5 m)
BT/GPS [11,12,13]	ease-of-use(smartphone)	low accuracy, low robustness
Camera/Image [18,19,20]	high accuracy	privacy concern
Magnetic field [25]	high accuracy/robustness	portability

**Table 2:** Hardware improvement space for next generation of the prototype

Hardware	Coil Size (cm)	PCB Size (cm)	PCB Components Number	PCB Components Density (%) ^1^	Estimation of Minimised PCB Size (cm)
Transmitter	0.8 (r) × 2.0 (h)	7.8 × 5.0 × 1.0	115, of which 82 are passive components ^2^	8.11	4.1 × 3.0 × 1.6 (estimated with density of 50%) ^3^
Receiver	1.8 × 1.8 × 1.8	4.9 × 4.2 × 1.0	349, of which 257 are passive components	14.55	(integrated into transmitter board)
Battery		7.2 × 3.5 × 2.5			5.4 × 3.5 × 0.6 (a common smartphone battery)
Overall		16 × 6.5 × 6.0			5.4 × 3.5 × 4.1

${}^{1}$ The density was calculated by dividing all components’ footprints size by the PCB size, representing the components occupied area in each square centimeter of the PCB. The density of on-board connections and wires are not considered in the table, since their layout problems can be easily addressed by increasing the layer from four to six, or even eight; ${}^{2}$ Passive components are single units, like resistors, capacitors, which have a small footprint of 1.0 × 0.5 or 1.6 × 0.8 mm; ${}^{3}$ This components density is common for a DFM (Design for manufacturability) [36] in current consumer electric devices. For example, in the latest generations of smartphones, the component to board footprint ratio is approximately above 70% [37]. This can be further increased by new innovative PCB technologies [38].

**Table 3:** Performance of the curve-fitting distance estimation approach.

Actual Distance (m)	0.5	0.75	1.0	1.25	1.50	1.75	2.0
Mean of Error (cm)	−1.3	1.9	1.4	−0.24	−2.9	−5.6	−6.7
Standard Deviation of Error (cm)	0.08	0.12	0.25	0.45	0.98	1.73	8.3

## Abbreviations

The following abbreviations are used in this manuscript:

LFSR	linear-feedback shift register
RSSI	Received Signal Strength Indicator
BT	Bluetooth
RF	Radio Frequency
ID	Identifier
GPS	Global Positioning System
TDMA	Time-Division Multiple Access
